# YTHDF1’s Regulatory Involvement in Breast Cancer Prognosis, Immunity, and the ceRNA Network

**DOI:** 10.3390/ijms25031879

**Published:** 2024-02-04

**Authors:** Wenting Luo, Youjia Zhou, Jiayang Wang, Keqin Wang, Qing Lin, Yuqiu Li, Yujie Xie, Miao Li, Jie Wang, Lixia Xiong

**Affiliations:** 1 Department of Pathophysiology, Medical College, Nanchang University, 461 Bayi Road, Nanchang 330006, China; 2 Second Clinical Medical College, Nanchang University, Nanchang 330006, China; 3 First Clinical Medical College, Nanchang University, Nanchang 330006, China; 4 Queen Mary School, Nanchang University, Nanchang 330006, China; 5 College of Pharmacy, Nanchang University, Nanchang 330006, China; 6 Key Laboratory of Functional and Clinical Translational Medicine, Xiamen Medical College, Fujian Province University, Xiamen 361023, China

**Keywords:** breast cancer, m6A, YTHDF1, prognosis, immunity, ceRNA

## Abstract

YTH N^6^-methyladenosine RNA binding protein 1 (YTHDF1), an m6A reader, has a role in the development and progression of breast cancer as well as the immunological microenvironment. The networks of competing endogenous RNA in cancer have received much attention in research. In tumor gene therapy, the regulatory networks of m6A and competing endogenous RNA are increasingly emerging as a new route. We evaluated the relationship between the YTHDF1 expression, overall survival, and clinicopathology of breast cancer using TCGA, PrognoScan, and other datasets. We used Western blot to demonstrate that YTHDF1 is substantially expressed in breast cancer tissues. Furthermore, we explored YTHDF1′s functions in the tumor mutational burden, microsatellite instability, and tumor microenvironment. Our findings indicate that YTHDF1 is a critical component of the m6A regulatory proteins in breast cancer and may have a particular function in the immunological microenvironment. Crucially, we investigated the relationship between YTHDF1 and the associated competitive endogenous RNA regulatory networks, innovatively creating three such networks (Dehydrogenase/Reductase 4-Antisense RNA 1-miR-378g-YTHDF1, HLA Complex Group 9-miR-378g-YTHDF1, Taurine Up-regulated 1-miR-378g-YTHDF1). Furthermore, we showed that miR-378g could inhibit the expression of YTHDF1, and that miR-378g/YTHDF1 could impact MDA-MB-231 proliferation. We speculate that YTHDF1 may serve as a biomarker for poor prognosis and differential diagnosis, impact the growth of breast cancer cells via the ceRNA network axis, and be a target for immunotherapy against breast cancer.

## 1. Introduction

In 2023, the latest data from the International Agency for Research on Cancer (IARC) revealed that breast cancer incidence rates surpassed lung cancer, making them the most common type of malignant tumors globally [1]. Currently, effective treatments for advanced or triple-negative breast cancer are limited, and the prognosis is poor. More research is needed to better understand the potential mechanisms behind the onset and progression of breast cancer [2]. Existing research has demonstrated that the occurrence and development of breast cancer entail intricate interactions between epigenetics and transcription in addition to genetic alterations [3]. However, it is currently difficult to pinpoint the exact molecular mechanism through which breast cancer is caused by epigenetic alterations [4].

Epigenetics regulates the expression of inherited genes without modifying the DNA sequence. Various forms of epigenetic modifications, such as DNA and RNA methylation, histone modification, chromatin remodeling, and non-coding RNA control, play a significant role in this process [5].

Numerous investigations have revealed that N6 methyladenine alteration happens in eukaryotes as well as prokaryotic bacteria, owing to the quick development of specialized antibodies and high-throughput sequencing [6]. One of the most frequent modifications in eukaryotic mRNA is the N6-methyladenosine (m6A) alteration, which has an impact on mRNA splicing, output, localization, translation, attenuation, and stability [7,8,9,10]. Three m6A regulators [11,12,13,14], known as the “writer”, “eraser”, and “reader”, are involved in this process. The “writer” includes methyltransferase-like 3 (*METTL3*), methyltransferase-like 14 (*METTL14*), Wilms tumor 1-associating protein (*WTAP*), RNA binding motif protein (*RBM15*), vir like m6A methyltransferase associated (*VIRMA*), and zinc finger CCCH-type containing 13 (*ZC3H13*). The “eraser” consists of fat mass and obesity-associated protein (*FTO*) and AlkB homolog 5, RNA demethylase (*ALKBH5*), while the “reader” encompasses YTH domain containing (*YTHDC1*), YTH domain containing (*YTHDC2*), YTH N6-Methyladenosine RNA binding protein 1 (*YTHDF1*), YTH N6-Methyladenosine RNA binding protein 2 (*YTHDF2*), and heterogeneous ribonucleoprotein particle (*HNRNP*). The task of the “writer” is to initiate the synthesis of m6A. The methylation of the target mRNA can be removed by the “eraser”. Through decoding m6A methylation and generating functional signals, the “reader” determines the fate of the targeted RNA and primarily regulates the regulation of m6A modification in gene expression [15,16]. The latest evidence has shown that m6A modification is crucial to many regular biological functions, particularly tumor immunity [17,18]. Furthermore, aberrant m6A modification and its related regulatory proteins—m6A writer, eraser, and reader proteins—have been found to play crucial roles in cancers such as leukemia [19], breast cancer [20], and liver cancer [21,22,23]. The regulatory mechanism and biological function of m6A have become a focus of RNA study. Still, more research is needed to fully comprehend how different biological processes are affected by m6A and how essential it is for m6A to perform a variety of functions in a range of circumstances [21].

The human body has an abundance of reading proteins from the YTH domain family, including YTHDF1-3, YTHDC1, and YTHDC2. Their involvement in nearly every stage of the mRNA metabolism process is noteworthy since they are crucial in regulating the cell fate of m6A-modified mRNA [24]. YTHDF1 and YTHDF3 are frequently amplified, which is associated with poor prognosis in breast cancer patients [25]. Following recent research, YTHDF1 has been intimately linked to the development and spread of several malignancies. According to research, YTHDF1’s major function is to promote m6A mRNA’s ribosome assembly and interaction with initiation factors, which speeds up m6A mRNA’s translation [26]. m6A promotes breast cancer lung metastasis by increasing the stability of the KRT7-AS/KRT7 mRNA duplex and translation of KRT7, where YTHDF1/eEF-1 is involved in FTO-regulated KRT7 mRNA translational extension [27]. YTHDF1 is closely associated with the development and progression of tumors. For instance, YTHDF1 participates in the m6A demethylase ALKBH5 pathway, contributing to the growth of lung cancer [28]. In colorectal cancer, YTHDF1 is considered to be an oncogene that has a direct effect on the aggressive character of an illness [29]. The overexpression of YTHDF1 in breast cancer indicates a bad prognosis [30]. Other studies have shown that YTHDF1 plays an important role in cancer development and long-lasting, novel, antigen-specific anti-tumor immunity [26]. In addition, YTHDF1 inhibits the function of anti-tumor immune cells in the tumor microenvironment (TME) by influencing the degree of the infiltration of CD8^+^ T cells and natural killer cells. Blocking YTHDF1 can reactivate the suppressed anti-tumor immunity and synergistically improve the therapeutic effect of anti-PD-L1 inhibitors [31]. Existing studies point out that YTHDF1 may be a potential prognostic and immunotherapeutic biomarker.

The m6A and ceRNA regulatory network represents a new trend in oncogene therapy. m6A-related mRNAs and lncRNAs can not only function as distinct potential targets for customized therapy in a variety of cancers, but also can predict prognosis in a variety of tumors [32]. It has been demonstrated that the miR-577/POSTN axis controls the downstream ILK/AKT/mTOR signaling pathway through the METTL3-mediated overexpression of lnc00520 to accelerate the development of breast cancer [33]. The YTHDF1/FOXM1 regulatory pathway contributes to metastasis and growth of breast cancer through regulating PKM2 to affect the glycolysis and progression of breast cancer [34]. Tumor hypoxia can induce HIF1, which post-transcriptionally inhibits the expression of miR-16-5p and promotes the expression of YTHDF1, which sequentially enhances tumor glycolysis via the up-regulation of PKM2, ultimately increasing the tumorigenic and metastatic potential of breast cancer cells [35]. Tan et al. [36] discovered that circBACH2 can function as a sponge molecule for miR-944 to modulate HNRNPC expression, which in turn speeds up the growth of breast cancer via the MAPK signaling pathway. Nevertheless, the mechanisms behind the interactions between m6A alterations and lncRNAs that impact the progression of breast cancer remain poorly understood, and it is still unknown how these changes interact with lncRNAs in tumors [37].

In the context of this study, we aimed to compare the m6A-related gene expression levels and epigenetic alterations in breast cancer to further better understand its function in tumor immunity and ceRNA network regulation. In this work, we examined the genetic alterations, functional expression levels, and contribution of YTHDF1 to the development of breast cancer. This might be particularly important for immunological penetration, immunomodulation, and immune checkpoints. YTHDF1 is highly expressed in breast cancer and could be a reliable biomarker for breast cancer’s poor prognosis and tumor immunity. Moreover, miR-378g was able to suppress YTHDF1 expression, miR-378g/YTHDF1 impacted MDA-MB-231 cell proliferation, and YTHDF1 was also engaged in the regulation of the ceRNA network. In general, we concluded that m6A regulators are critical agents in the malignant progression of breast cancer, and the high expression of YTHDF1 may have specific roles in breast cancer prognosis and tumor immunity, as well as participate in ceRNA network regulation, which is anticipated to be a potential target in breast cancer immunotherapy.

## 2. Results

### 2.1. Transcriptional and Epigenetic Alterations of Major m6A Regulators in Breast Cancer Tissue

**Figure 1:**
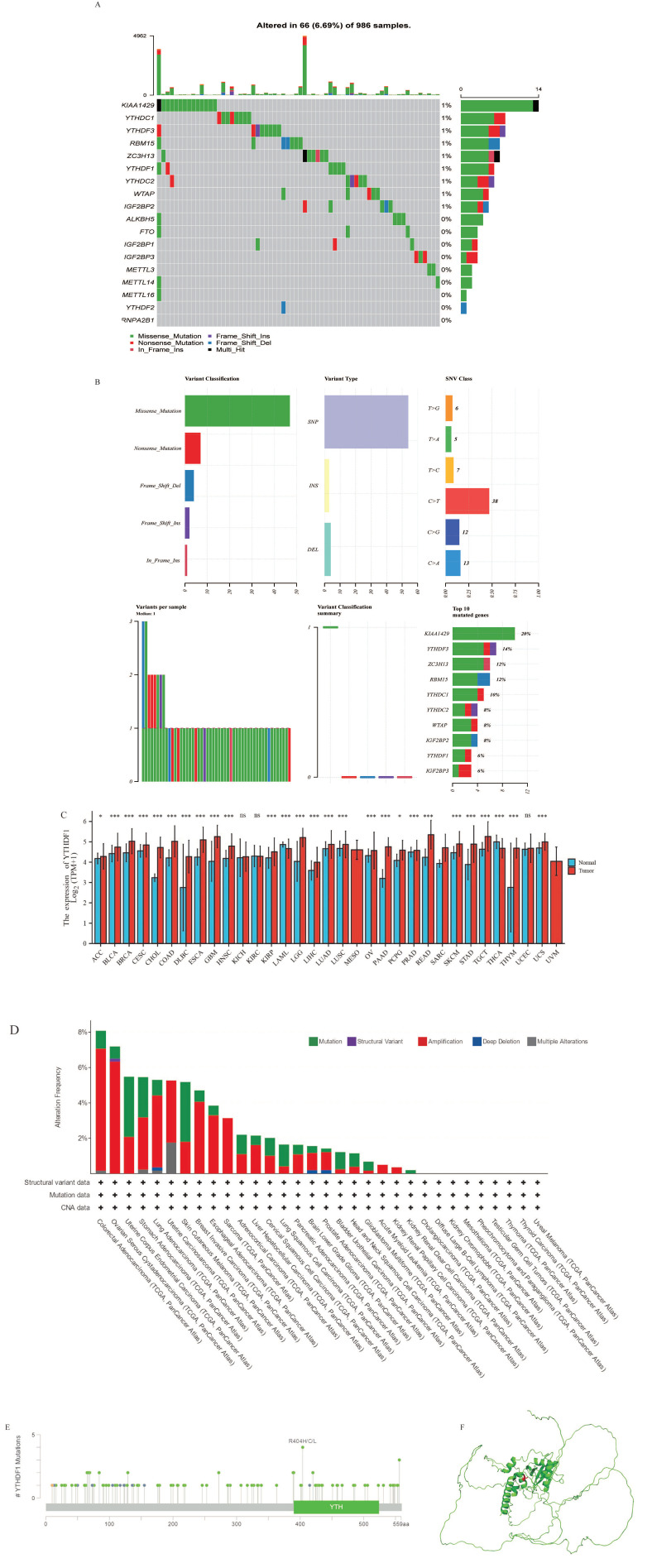
Gene expression profiles of 18 m6A regulators in breast cancer and mutation characteristics of YTHDF1 in different TCGA tumors. (**A**) Waterfall diagram of gene mutation analysis of 18 m6A regulators in 986 TCGA patients. (**B**) Summary map of m6A regulatory gene mutations. The 10 most frequently mutated m6A regulators were KIAA1429, ZC3H13, YTHDF3, YTHDC1, HNRNPA2B1, RBM15, YTHDF1, YTHDC2, WTAP, and IGF2BP2. (**C**) Expression patterns of YTHDF1 in various tumors. ACC: adrenocortical carcinoma; BLCA: bladder urothelial carcinoma; BRCA: breast invasive carcinoma; CESC: cervical squamous cell carcinoma and endocervical adenocarcinoma; CHOL: cholangiocarcinoma; COAD: colon adenocarcinoma; DLBC: lymphoid neoplasm diffuse large B-cell lymphoma; ESCA: esophageal carcinoma; GBM: glioblastoma multiforme; HNSC: head and neck squamous cell carcinoma; KICH: kidney chromophobe; KIRC: kidney renal clear cell carcinoma; KIRP: kidney renal papillary cell carcinoma; LAML: acute myeloid leukemia; LGG: brain lower grade glioma; LIHC: liver hepatocellular carcinoma; LUAD: lung adenocarcinoma; LUSC: lung squamous cell carcinoma; MESO: mesothelioma; OV: ovarian serous cystadenocarcinoma; PAAD: pancreatic adenocarcinoma; PCPG: pheochromocytoma and paraganglioma; PRAD: prostatic adenocarcinoma; READ: rectum adenocarcinoma; SARC: sarcoma; SKCM: skin cutaneous melanoma; STAD: stomach adenocarcinoma; TGCT: testicular germ cell tumors; THCA: thyroid carcinoma; THYM: thymoma; UCEC: uterine corpus endometrial carcinoma; UCS: uterine carcinosarcoma; UVM: uveal melanoma. NS, not significant; * *p* < 0.05; *** *p* < 0.001. We analyzed the characteristics of YTHDF1 mutations in TCGA tumors using the cBioPortal tool. The frequency of mutation type (**D**) and mutation site (**E**) are shown, (**E**) yellow dot: splice site, green dot: missense mutation, blue dot: frame shift del. (**F**) The 3D structure diagram of YTHDF1(marked in green), where the red-labeled sites in the figure are the R404 sites in the YTHDF1 protein structure that are prone to missense mutations.

In our study, we selected a total of 18 m6A regulators. At first, we evaluated 986 breast cancer samples in the TCGA database and found that 66 of them (6.69%; Figure 1A) were mutated. The 10 most frequently mutated m6A regulators were KIAA1429, ZC3H13, YTHDF3, YTHDC1, HNRNPA2B1, RBM15, YTHDF1, YTHDC2, WTAP, and IGF2BP2, and YTHDF1 also had a high mutation frequency (8%; Figure 1B), which laid the foundation for our subsequent study. We further observed the expression pattern of YTHDF1 in different diseases, as shown in Figure 1C. Compared with normal tissues, YTHDF1 was significantly up-regulated in 26 diseases, including breast cancer, and significantly down-regulated in acute myeloid leukemia (LAML) and thyroid cancer (THCA). These findings indicate that YTHDF1 is aberrantly expressed in different disease types, and mostly in an overexpressed manner. The mutation frequency of YTHDF1 in breast tumors was 4.7%, with the “amplified” type of CNV being the main type of mutation in the breast cancer cases, occurring at a frequency of alteration of about 4% (Figure 1D). Figure 1E depicts the kinds and locations of the YTHDF1 gene mutations. With notable sites for the R404H/C/L mutations, we identified missense mutations in YTHDF1 as the predominant form of genetic changes in breast cancer. The 3D structural map of YTHDF1 is displayed in picture F, and the red-labeled sites in the figure are the R404 sites that are prone to missense mutations in the YTHDF1 protein structure (Figure 1F).

### 2.2. YTHDF1 Is Up-Regulated in Breast Cancer and Associated with Poor Prognosis

**Figure 2:**
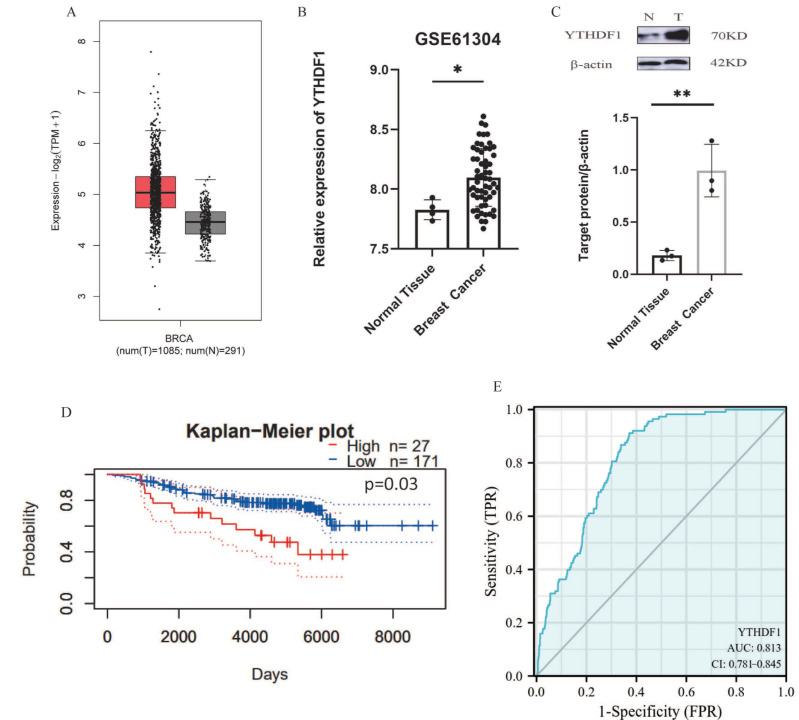
Expression of YTHDF1 in breast cancer and prognosis. (**A**) Up-regulation of YTHDF1 expression in breast cancer tissues in the TCGA database. (**B**) Up-regulation of YTHDF1 expression in breast cancer tissues in the GEO database. (**C**) Western blot analysis showed that YTHDF1 was highly expressed in breast cancer. (**D**) Up-regulation of YTHDF1 predicts poor prognosis of breast cancer. The dotted curves represent the 95% confidence interval. The red solid curve represents the group with low YTHDF1 expression, and the blue solid curve represents the group with high YTHDF1 expression. (**E**) ROC curve of YTHDF1. The curve showed that the AUC value of YTHDF1 was 0.813 (95% CI: 0.781–0.845). * *p* < 0.05; ** *p* < 0.01.

Initially, we used bioinformatics tools (GEPIA, http://gepia.cancer-pku.cn/, accessed on 8 June 2022.) to analyze the expression of YTHDF1 in breast cancer tissues. The results revealed that YTHDF1 was overexpressed in breast cancer patients (Figure 2A). Furthermore, we investigated in detail the transcriptional patterns of 58 breast cancer tissues and 4 non-tumorigenic (NT) breast tissues from the GEO database (GSE61304). The findings showed that, in comparison to normal breast tissues, the m6A reading protein YTHDF1 mRNA was considerably increased in breast cancers (Figure 2B). Using Western blot on previously obtained samples of breast cancer and normal breast tissue, we verified that YTHDF1 was significantly expressed in breast cancer (Figure 2C). In addition, we noticed that its overexpression in breast cancer was associated with a poor prognosis through utilizing the online data platform Kaplan–Meier (http://kmplot.com/analysis/, accessed on 8 June 2022.), which is based on meta-analysis to detect biological indicators of survival for cancer research (Figure 2D). A ROC curve study (Figure 2E) was performed to explore the efficiency of YTHDF1 in distinguishing breast cancer samples (*n* = 1198) from normal samples (*n* = 113). The AUC was 0.813 (95% CI: 0.781–0.845). To summarize, the increased expression of YTHDF1, one of the main readers in m6A, may be a significant factor in the advancement and a useful indicator of predicting breast cancer and its prognosis.

### 2.3. PPI Network and Functional Annotations

**Figure 3:**
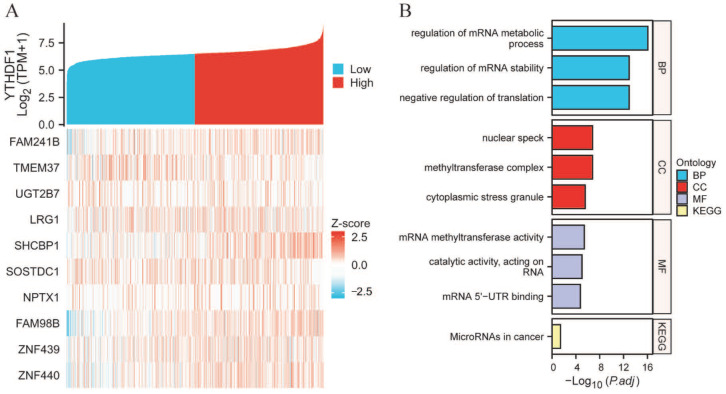
PPI network analysis and functional analysis. (**A**) Network of YTHDF1 and its top ten co-expressed genes. (**B**) Functional enrichment of YTHDF1 and its top ten co-expressed genes.

We created PPI networks to study the genes interacting with YTHDF1 in breast cancer (*n* = 1226) and their potential roles, and we carried out GO and KEGG correlation analyses using R and STRING to expand our understanding of the role of YTHDF1 in breast cancer. The top ten genes that were co-expressed most strongly with YTHDF1 were identified as being *FAM241B*, *TMEM37*, *UGT2B7*, *LRG1*, *SHCBP1*, *SOSTDC1*, *NPTX1*, *FAM98B*, *ZNF439*, and *ZNF440* (Figure 3A). The enrichment analysis revealed that these genes were collectively involved in processes such as mRNA metabolism control, stability regulation, and catalytic RNA activity, and changes in this process were connected to tissue components such as nuclear speck, methyltransferase complexes, and cytoplasmic stress granules (Figure 3B).

### 2.4. Analysis of YTHDF1’s Contribution to Breast Cancer Tumor Immunity

**Figure 4:**
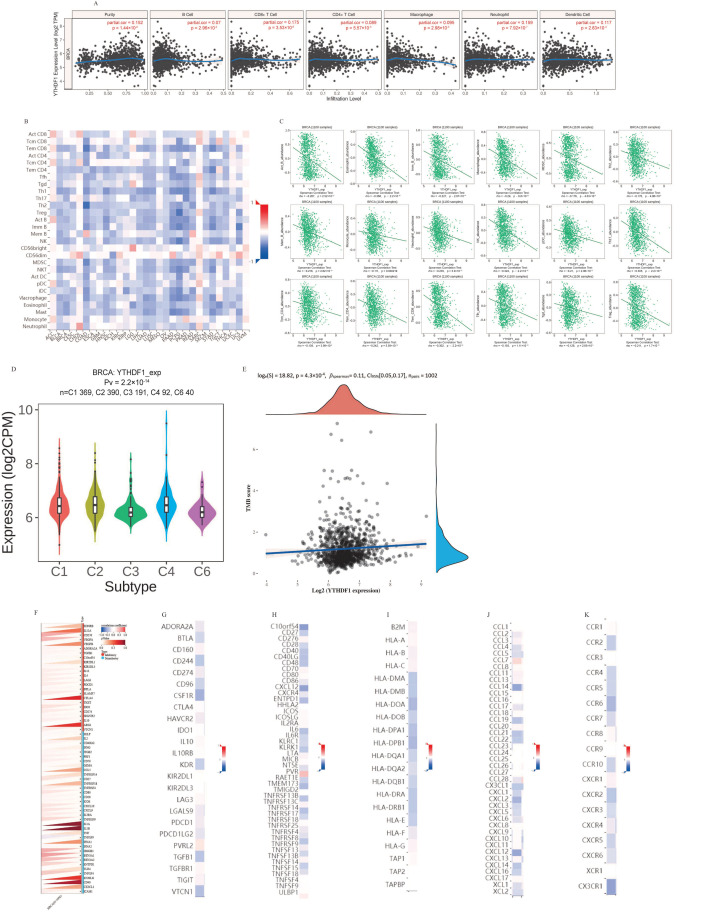
Analysis of the role of YTHDF1 in breast cancer immunity. (**A**) YTHDF1 expression was positively correlated with tumor purity and was also correlated with dendritic cells, CD4^+^ T cells, CD8^+^ T cells, neutrophils, and macrophages in breast cancer. (**B**) The relationship between YTHDF1 expression and the expression of 28 TILs in human cancer. (**C**) Correlation between YTHDF1 and the abundance of 18 TIL cells. (**D**) The relationship between YTHDF1 and immune subtypes. Spearman’s correlation analysis of TMB (**E**) and YTHDF1 gene expression. In the figure, the abscissa represents the gene expression distribution, the ordinate represents the TMB score distribution, and the density curve on the right represents the TMB score distribution trend. The upper density curve shows the trend of gene expression distribution. The uppermost value represents the *p* value of correlation, correlation coefficient, and correlation calculation method. (**F**) Correlation between YTHDF1 and immune checkpoint, * *p* < 0.05. The relationship between YTHDF1 and five immune molecules (**G**) immune stimulator, (**H**) immune suppressor, (**I**) MHC, (**J**) chemokine, and (**K**) chemokine receptor in breast cancer.

A rising number of studies have demonstrated the significant impact of tumor-infiltrating lymphocytes on the advancement of cancer and their influence on the outlook for individuals with breast cancer. Therefore, we analyzed the correlation between YTHDF1 expression and six types of tumor-infiltrating immune cells (TIICs) in the TIMER database. YTHDF1 expression was linked with tumor purity (r = 0.152, *p* = 1.44 × 10^−6^), B cells (r = 0.07, *p* = 2.96 × 10^−2^), CD8^+^ T cells (r = 0.175, *p* = 3.53 × 10^−8^), CD4^+^ T cells (r = 0.089, *p* = 2.96 × 10^−2^). *p* = 5.57 × 10^−3^), macrophages (r = 0.095, *p* = 2.98 × 10^−3^), neutrophils (r = 0.159, *p* = 7.92 × 10^−7^), and dendritic cells (r = 0.117, *p* = 2.83 × 10^−4^) (Figure 4A). The findings revealed that YTHDF1 had a favorable relationship with tumor purity and these TIICs. Figure 4B depicts the assessment of the relationship between YTHDF1 and the expression of 28 TILs in human malignancies using TISIDB data (Figure 4B). Specifically, there was a negative correlation between YTHDF1 and 18 TILs (Figure 4C).

Furthermore, we explored how YTHDF1 expression affected breast cancer immunity and molecular subtypes using the TISIDB website. As shown in Figure 4D, immune subtypes were classified into six types including C1 (wound healing), C2 (IFN-γ dominant), C3 (inflammation), C4 (lymphocyte depletion), C5 (immune calm), and C6 (TGF-b dominant). The results showed that the expression of YTHDF1 differed with the different immune subtypes of breast cancer (*p* = 2.2 × 10^−14^). In order to better understand the role of YTHDF1 in the immune mechanism of TME, we analyzed the correlation between YTHDF1 expression and TMB. In particular, we selected 1101 RNA SEQ data and the associated clinical data related to breast cancer from the TCGA dataset. Spearman’s correlation analysis was used to describe the correlation between quantitative variables without normal distribution. YTHDF1 was positively correlated with TMB (Figure 4E) .

Clinically, immune checkpoint blockade has brought significant benefits against malignancies. However, the efficacy of immune checkpoint blockade in breast cancer is controversial. There is a limited benefit population in the clinical studies that have examined the impact of PD1/PDL1 checkpoint inhibitors on breast cancer. Therefore, we acquired a collection of uniformly normalized breast cancer data (*n* = 1092) from the UCSC database. Additionally, 60 immune checkpoint pathway genes, including 36 stimulating and 24 inhibitory genes, were extracted from the breast cancer sample data, and all the normal samples were filtered. These data contained the ENSG00000149658 (YTHDF1) gene expression data. For every expression value, log2 (x + 0.001) alterations were also carried out. Subsequently, we evaluated the Pearson correlation between ENSG00000149658 (YTHDF1) and the immune pathway marker genes (Figure 4G). After that, we analyzed the correlation between YTHDF1 expression in breast cancer and immune stimulatory molecules (Figure 4H), immunosuppressive molecules (Figure 4I), and MHC molecules (Figure 4J), chemokines (Figure 4K), and chemokine receptors (Figure 4L) using the TISIDB database. The strongest positive correlations were observed between YTHDF1 and the immunostimulatory molecule PVRL2, the immunosuppressive molecule RAET1E, the MHC molecule TAP1, the chemokine CCL7, and the chemokine receptor CCR8, respectively.

### 2.5. Analysis of YTHDF1-Associated ceRNA Network in Breast Cancer

**Figure 5:**
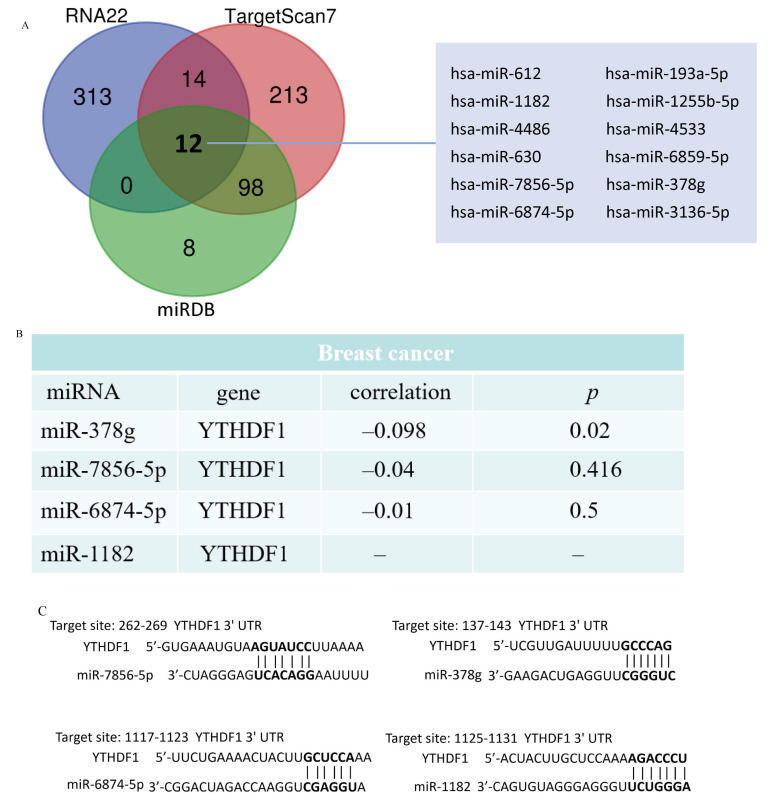
Prediction of YTHDF1 target miRNAs in breast cancer. (**A**) Venn diagram showing the prediction results of YTHDF1 targets from RNA22, TargetScan7, and miRDB databases. (**B**) Analysis of the correlation between YTHDF1 and target miRNAs. (**C**) Prediction of potential binding sites of YTHDF1 to target miRNAs, potential binding sites of YTHDF1 to the corresponding miRNA are shown in bold.

Increasing evidence suggests that the lncRNA–miRNA–mRNA ceRNA network plays a critical role in a variety of human cancers, so we set out to analyze and build a ceRNA network involving YTHDF1 in breast cancer. Using RNA22, TargetScan7, and miRDB databases, we analyzed and predicted 339, 337, and 118 YTHDF1 target miRNAs, respectively. The Venn diagrams depicted the predicted results of the YTHDF1 target miRNAs in RNA22, TargetScan7, and miRDB and the three databases jointly predicted 12 target miRNAs (Figure 5A, Table S2). To choose miRNAs that work better under ceRNA circumstances, we also examined the relationship between the target miRNA expression and YTHDF1 expression. The correlation analysis demonstrated that the expression levels of four target miRNAs, miR-7856-5p, miR-378g, miR-6874-5p, and miR-1182, were negatively linked with YTHDF1 (Figure 5B). TargetScan7 predicted the potential binding sites of YTHDF1 to these four target miRNAs (Figure 5C).

**Figure 6:**
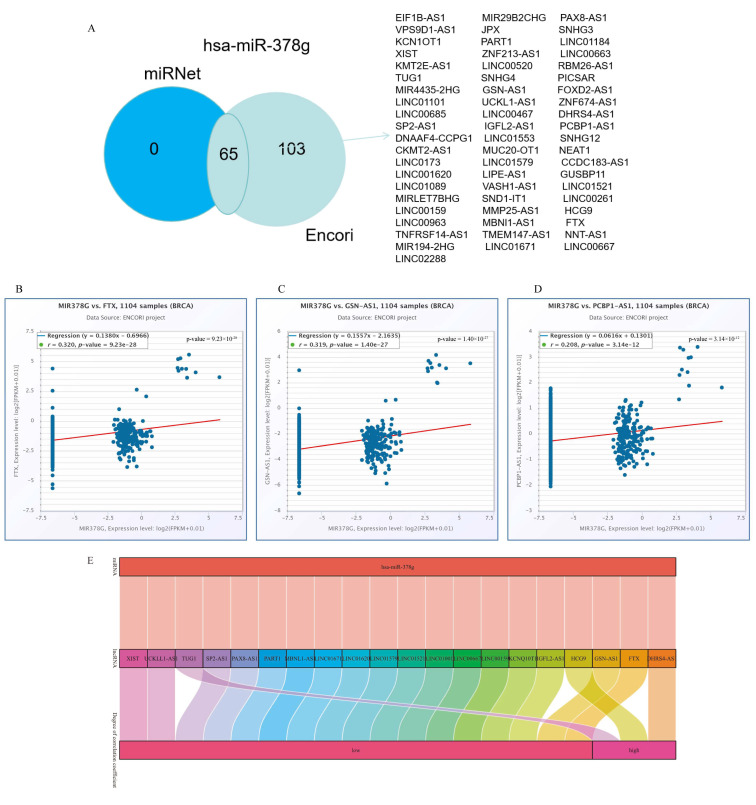
Prediction of lncRNA and ceRNA network construction in breast cancer. (**A**) Venn diagram showing the target lncRNAs of miR-378g. Correlations between miRNAs and target lncRNAs were analyzed using ENCORI (starBase v2.0) software. Scatter plots were used to show miRNA–lncRNAs with significant correlation. (**B**) Correlation between miR-378g and FTX. (**C**) Correlation between miR-378g and GSN-AS1. (**D**) Correlation between miR-378g and PCBP1-AS1. (**E**) Sankey diagram showing the lncRNA–miRNA–mRNA (YTHDF1) regulatory network consistent with the ceRNA hypothesis.

To further forecast the lncRNAs that may bind to four target miRNAs (miR-7856-5p, miR-378g, miR-6874-5p, miR-1182), we used the online databases miRNet 2.0 and ENCORI. The results revealed that only one miRNA (miR-378g) existed with lncRNAs, and the Venn diagram shows the predicted results from both databases (Figure 6A). Based on the ceRNA network hypothesis, a negative correlation exists between the lncRNAs and miRNAs. As a result, we analyzed the correlation between the expression of the target lncRNAs and miR-378g in breast cancer using the ENCORI database. Twenty lncRNAs were found to be negatively connected with miR-378g, while three target lncRNA expression levels, FTX (Figure 6B), GSN-AS1 (Figure 6C), and PCBP1-AS1 (Figure 6D), were more strongly negatively correlated with miR-378g. According to the ceRNA hypothesis, there is an inverse relationship between miRNA and lncRNA or mRNA, so we constructed 20 pairs of ceRNA networks based on the results of the correlation analysis, among which three pairs of ceRNA networks (DHRS4-AS1-miR-378g-YTHDF1, HCG9-miR-378g-YTHDF1, TUG1- miR-378g-YTHDF1) showed a more significant correlation (Figure 6E).

### 2.6. miR-378g Inhibits YTHDF1 Expression and Proliferation of MDA-MB231 Cells

**Figure 7:**
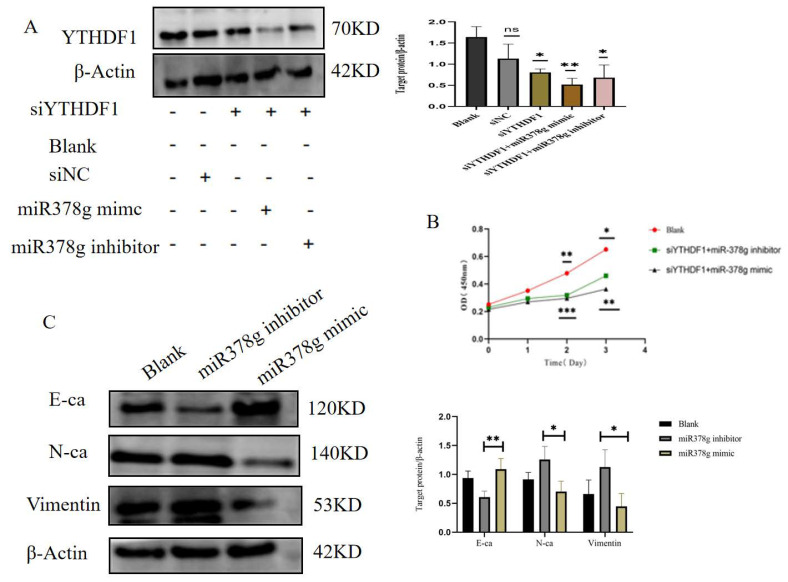
miR-378g inhibits YTHDF1 expression and proliferation of MDA-MB231 cells. (**A**) Western blot results indicated that YTHDF1 expression was up-regulated in response to down-regulated miR-378g expression. (**B**) CCK-8 results showed that up-regulation of miR-378g expression delayed the proliferation rate of MDA-MB-231 cells in knockdown of YTHDF1 after 48 h and 72 h. (**C**) Western blot analysis of EMT expression of miR378g inhibitors and mimics in breast cancer cells. (ns: no significance; * *p* < 0.05, ** *p* < 0.01, *** *p* < 0.001).

One common link between the three lncRNA/miRNA/mRNA axes we built was miR-378g with YTHDF1. Therefore, we investigated the relationship between miR-378g and YHTDF1 in more detail. To confirm that miR-378g has an impact on YTDF1 expression, we used miR-378g mimics and miR-378g inhibitors to transfect MDA-MB-231 cells knocked down with YTHDF1. The Western blot results indicated that YTHDF1 expression was up-regulated in response to down-regulated miR-378g expression. When miR-378g expression was increased, YTHDF1 expression was reduced (Figure 7A). Further, we investigated the miR-378g/YTHDF1 regulation of MDA-MB-231 cell proliferation in vitro. The cck-8 results showed that the up-regulation of miR-378g expression delayed the proliferation rate of the MDA-MB-231 cells in the knockdown of YTHDF1 after 48 h and 72 h, whereas, the knockdown of YTHDF1 had a significant inhibitory effect on the rate of MDA-MB-231 cell proliferation, regardless of miR-378g expression (Figure 7B). These results demonstrate that miR-378g can interact with YTHDF1, the overexpression of miR-378g can inhibit YTHDF1 expression, and miR-378g/YTHDF1 interaction can regulate the proliferation of MDA-MB-231 cells. Finally, we verified the effect of miR378g mimics and inhibitors on MDA-MB-231 breast cancer cells by detecting EMT indicators, which was consistent with the results of the CCK8 experiment.

## 3. Discussion

A new approach for immunotherapy and the development of targeted drugs in oncology is emerging as a result of extensive study on m6A RNA methylation, which has revealed its supporting role in the initiation and progression of cancers [38]. Aberrant m6A modifications regulate immune cell infiltration, pro-tumor inflammation, immunosuppression, immunosurveillance, and anti-tumor immune responses in a variety of malignancies [39]. YTH structural domain-containing proteins (YTHDFs and YTHDCs) are members of a highly conserved family among eukaryotic proteins that are connected to various forms of mRNA metabolism and partake in physiological activities [40]. In tumor immunity, YTHDF1-deficient mice have increased dendritic cell capacity to present tumor antigens, resulting in increased immune response initiation by T cells and a stronger anti-tumor CD8+ T cell response [31]. Furthermore, it was discovered that YTHDF1 promotes NSCLC cell proliferation and xenograft carcinogenesis via modulating the translation efficiency of various immunological checkpoints [41]. The inhibitory factor HINT2 mRNA translation is stimulated by YTHDF1 in melanoma, and its absence enhances the anti-melanoma immune response [42]. These findings indicate that YTHDF1 is likely to be a promising target for anti-tumor immunotherapy.

In this study, we analyzed 986 samples from the TCGA database and found a high frequency of mutations in YTHDF1. The most common types of genetic alterations were missense mutations and the "amplified" type of CNV. Moreover, we found that YTHDF1 was expressed abnormally in a number of cancers, primarily in an overexpressed form. We then looked into the expression of YTHDF1 in breast cancer tissues and its relationship to prognosis. A Western blot showed that YTHDF1 was highly expressed in breast cancer tissues. And we found that high YTHDF1 mRNA expression was associated with a shorter overall survival in breast cancer based on Kaplan–Meier curves and univariate analysis. In addition, we deduced that YTHDF1 may be employed as a biomarker for breast cancer with a bad prognosis.

In order to better understand the probable mechanism of YTHDF1 in breast cancer, we undertook gene analysis of YTHDF1 co-expression. We discovered that the top 10 genes with the highest association to YTHDF1 co-expression were FAM241B, TMEM37, UGT2B7, LRG1, SHCBP1, SOSTDC1, NPTX1, ZNF439, and ZNF440. The enrichment study revealed that they work together to regulate biological processes such mRNA metabolism, stability, and catalytic RNA activity. Changes in this process are linked to tissue components like nuclear speckles, methyltransferase complexes, and cytoplasmic stress granules. Breast cancer cells are more susceptible to the PARP inhibitor Olaparib, adriamycin, and cisplatin when YTHDF1 is knocked down, because it facilitates S-phase entrance, DNA replication, and DNA damage repair [43].

In the next section, we discussed the expression of YTHDF1 in different immune and molecular subtypes of breast cancer. The results showed that YTHDF1 expression varied considerably among immunological and molecular subtypes. It raises the prospect that YTHDF1 might serve as a biological marker for the detection of breast cancer and have a specific role in immunomodulatory processes. TILs in the TME take a pivotal role in cancer progression that affects the prognosis of breast cancer patients [44]. In our study, we discovered that immune cell infiltration, including B cells, CD8+ T cells, CD4+ T cells, macrophages, neutrophils, and dendritic cells, was highly linked with YTHDF1 expression patterns in the TIMER database, which was similar to the results of a previous study [45]. Additionally, we examined the 28 TILs in the TISIDB database to determine their relationships with YTHDF1 expression, and we observed that there was a negative correlation between YTHDF1 and the abundance of 18 of these TILs. The downstream targets of YTHDF1 may be related to MYC signaling regulation and T-cell differentiation, and YTHDF1 amplification resulted in reduced immune cell infiltration and significantly worse clinical features in breast cancer patients [46].

ICP genes significantly affect immune cell infiltration and immunotherapy [47]. Tumor-specific neoantigens serve as useful targets for cancer immunotherapy and are crucial indicators for determining the effectiveness of immune checkpoint blockade treatments [9]. YTHDF1 deletion in dendritic cells was previously found to improve anti-tumor immunity and slow tumor development in a mouse model [31]. In order to comprehend the potential role of YTHDF1 in immunotherapy, we investigated the relationship between YTHDF1 expression and breast cancer immune checkpoint genes. The findings showed that YTHDF1 expression was positively linked with the majority of the immunological checkpoints relevant to breast cancer, indicating that YTHDF1 is probably a target for treating breast cancer and determining the effectiveness of therapeutic interventions [48].

Immune molecules have a significant role in controlling the tumor response [49,50]. We noted a link between YTHDF1 expression in breast cancer and immunostimulatory molecules, immunosuppressive molecules, MHC molecules, chemokines, and chemokine receptors. Moreover, our study also revealed that YTHDF1 was favorably correlated with TMB. These findings add to the evidence that YTHDF1 and TME are closely related to breast cancer.

Since m6A is known to participate in lncRNA-mediated ceRNAs, ceRNA networks are assumed to be important in the pathogenesis of cancer. Such networks are considered to work primarily through the competitive binding of lncRNAs or circRNAs to miRNAs, which in turn affects the levels of mRNA expression [51]. In this research, we first predicted the target miRNAs that bind to YTHDF1 in breast cancer using RNA22, TargetScan7, and miRDB databases, respectively, and found that all three of these databases predicted a total of 12 potential upstream target miRNAs, with a subsequent correlation analysis finding that only four miRNAs (miR-7856-5p, miR-378g, miR-6874-5p, miR-1182) had a more significant negative correlation with YTHDF1. Further, we predicted the upstream lncRNAs of the four miRNAs mentioned above. Only one miRNA, miR-378g, existed in the upstream lncRNA. Therefore, we investigated the connection between miR-378g and YTHDF1 through in vitro tests. We discovered that miR-378g could suppress YTHDF1 expression and slow the rate at which MDA-MB-231 cells proliferated.

Through correlation analysis, we discovered that only three lncRNAs—DHRS4-AS1, HCG9, and TUG1—were strongly linked with miR-378g. In particular, we created three ceRNA network pairings in response to these findings. DHRS4-AS1 could be detected through the DHRS4-AS1/miR-522-5p/SOCS5 axis on cell proliferation, apoptosis, and cell cycle in hepatocellular carcinoma, which in turn regulates the development of hepatocellular carcinoma [52]. HCG9 can facilitate the proliferation of osteosarcoma cells by inhibiting miR-34b-3p [53]. TUG1 regulates the resistance of breast cancer cells to adriamycin by targeting binding to miR-9-5p and by acting on the downstream target eIF5A2 [54].

There is no denying that our study has certain limitations. Firstly, the majority of the data we used for our research in the study came from internet sources, necessitating further laboratory tests to confirm these conclusions. Secondly, while we believe that YTHDF1 expression may play an important role in the immune process of breast cancer, the correlation between YTHDF1 and tumor purity and TICs was not statistically significant. Additionally, our knowledge of the intrinsic mechanism is still limited, and more research is required to confirm YTHDF1’s potential involvement in breast cancer immunity. To increase their effectiveness in clinical applications, we also need to carry out further work on the functions and interactions of YTHDF1 with molecules including TMB and neoantigens.

## 4. Materials and Methods

### 4.1. Tissue Collection

Breast cancer samples from the First Affiliated Hospital of Nanchang University from February 2017 to February 2018 that were surgically removed and confirmed by pathological diagnosis were collected.

### 4.2. TCGA Database

YTHDF1 transcript expression data and corresponding clinical information were downloaded from the TCGA website (http://portal.gdc.cancer.gov/, accessed on 23 April 2022). In various cancer types, at least 5 samples were included in the normal group. The RNA-Seq gene expression data in FPKM format were converted to TPM format, then transformed to log2 for further analysis. All the data mentioned above were downloaded from the TCGA website, so the study did not require approval by the relevant ethics committee.

### 4.3. RNA Sequencing Data of YTHDF1 in Breast Cancer

The RNA-Seq expression data in breast cancer were downloaded from the TCGA database. The selected samples contained YTHDF1 expression data and relevant clinical information, including age, sex, TNM stage, and anatomical location of the tumor. mRNA expression data were expressed as mean ± standard deviation.

### 4.4. Protein–Protein Interaction (PPI) Network and Functional Enrichment Analysis

STRING (http://string-db.org, accessed on 29 May 2022) is an online database for searching protein–protein interactions. We used STRING to find co-expressed genes, then construct PPI networks for interaction scores. GO and KEGG pathway analysis were performed by the ClusterProfiler package and visualized by the “ggplot2” package.

### 4.5. Tumor Immune Estimation Resource (TIMER) Database

TIMER (http://cistrome.shinyapps.io/timer/, accessed on 10 June 2022) is a comprehensive online resource website for the systematic analysis of immune infiltration in various tumors. In the study, we used TIMER to determine the expression of YTHDF1 in breast cancer concerning six types of immune cells (B cells, CD4^+^ T cells, CD8^+^ T cells, neutrophils, macrophages, and dendritic cells). *p* values < 0.05 were considered statistically significant.

### 4.6. Tumor Immune System Interaction Database (TISIDB)

TISIDB (http://cis.hk/TISIDB/, accessed on 10 June 2022) is an online web-based database frequently used for tumor immune system interactions. In this work, we used TISIDB to discover the expression of YTHDF1 and tumor-infiltrating lymphocytes (TILs) in human cancers. Based on gene expression profiles, the relative abundance of tumor-infiltrating lymphocytes was inferred with the use of genomic variants. Spearman’s was used to examine the relevance of YTHDF1. Moreover, we also analyzed the correlation of YTHDF1 expression in breast cancer with immunostimulatory molecules, immunosuppressive molecules, major histocompatibility complex (MHC) molecules, chemokines, and chemokine receptors based on this database.

### 4.7. RMVar Database

RMVar (https://rmvar.renlab.org/, accessed on 20 August 2022) is an open-source online database involving functional variants of RNA modifications. It allows for a comprehensive analysis of RNA variants, such as m6A, m5C, m1A, etc.

### 4.8. PrognoScan Database

The PrognoScan database is a powerful online platform for assessing the correlation between gene expression and survival in different cancers [55]. In this report, we analyzed the correlation between YTHDF1 expression and OS in breast cancer using the selected dataset (GSE7390) from PrognoScan database.

### 4.9. Immune-Checkpoint-Related Database in Breast Cancer

Immune checkpoint signals can create an immunosuppressive environment. Reactivation of the immune system by immune checkpoint inhibitors to eradicate breast cancer has emerged as a promising therapeutic strategy [56]. We explored the association of YTHDF1 expression with immune checkpoints through the SangerBox website (http://sangerbox.com/Tool, accessed on 30 August 2022), a useful online platform for TCGA data analysis [57]. We downloaded standardized breast cancer data from the USCS database and further analyzed the correlation between the YTHDF1 gene and 60 immune checkpoint pathway genes.

### 4.10. Tumor Mutational Burden (TMB) Analysis

TMB is defined as the total number of somatic mutations per Mb base in the exon-coding region and is an emerging biomarker for determining the efficacy of tumor immunotherapy [58]. TMB is calculated as the total number of somatic mutations/target region size in mutations (Mb).

### 4.11. Functional Enrichment Analysis

“ClusterProfiler” package was applied to the analysis of GO and KEGG enrichment based on YTHDF1-related DEGs [59]. Adjusted *p*-values <0.05 were statistically significant.

### 4.12. Construction of ceRNA Network

RNA22 (https://cm.jefferson.edu/rna22/, accessed on 12 October 2022), TargetScan7 (https://www.targetscan.org/vert_72/, accessed on 26 October 2022), and miRDB (https://mirdb.org/, accessed on 12 October 2022) databases were used to predict YTHDF1 target miRNAs and analyze the correlation of YTHDF1 with target miRNAs. TargetScan7 online tool was used to predict the potential sites of YTHDF1 binding to target miRNAs. The miRNet2.0 (https://www.mirnet.ca/miRNet/home.xhtml, accessed on 27 October 2022) and ENCORI (https://starbase.sysu.edu.cn/index.php, accessed on 27 October 2022) online databases were used to further predict the association with miRNAs (miR-7856-5p, miR-378g, miR-6874-5p, miR-1182) bound to target lncRNAs, and correlations between the expression of both were analyzed using the ENCORI online database. The miRNA–mRNA and miRNA–lncRNA with negatively correlated expression levels were analyzed comprehensively to establish a critical lncRNA–miRNA–mRNA (YTHDF1) ceRNA network in breast cancer.

### 4.13. Cell Culture

The human breast cancer cell was purchased from Shanghai Cell Bank. The cells were cultured in DMEM high glucose (hyclone, Logan, UT, USA), supplemented with 10% fetal bovine serum (FBS) (BI, Kibbutz, Beit HaEmek, Israel) and 1% penicillin-streptomycin (Solarbio, Beijing, China) in a cultured environment of 37 °C constant temperature, 5% CO_2_ incubator.

### 4.14. Cell Transfection

Before transfection, 3.5 × 10^5^ cells were inoculated in each well of the six-well plate. When the cells had grown to about 60% of the bottom of the dish, Lipofectamine 3000 (Thermo Fisher, Waltham, MA, USA) and Opti-MEM serum-free medium (Gibco, Grand Island, NY, USA) were combined for YTHDF1 knockdown. In addition, miRNA mimics (miR-378g- mimics), miRNA inhibitors (miR-378g inhibitors), and siYTHDF1 were prepared as transfection complexes and added into 6 wells. The cells were transfected into the plate. The above siRNA was constructed by HanBiO (HanBiO, Shanghai, China), while the miRNA was constructed by Jima gene (Jima gene, Suzhou, China). The total protein was extracted from the cells 48 h later, and the transfection efficiency was determined by Western blot. These sequences are shown in Table S1.

### 4.15. Cell Proliferation Assay

The Cell Counting Kit-8 (CCK-8) (TransGen Biotech, Beijing, China) was used to detect the proliferation of MDA-MB-231, and the cells in the logarithmic growth phase that had been transfected in the early stage were used to inoculate 1000 cells per well in a 96-well plate. More than 4 duplicate wells were set in the group, and the zero-adjustment group only contained medium and CCK. Three 96-well plates were set for 24 h, 48 h, and 72 h. The cells were treated for the first 2 h, washed three times with sterile PBS, and then 90 μL of culture medium and 10 μL of CCK reagent were added to each well. Incubate in the incubator for 1 h, use a microplate reader to measure the OD value of each well at 450 nm and record, and the CCK value is the value obtained by subtracting the blank well from the measured value.

### 4.16. Western Blotting

Forty-eight hours after the transfection of MDA-MB-231 cells, cell lysates were collected using 100 μL of RIPA lysate (Applygen, Beijing, China) and 10 μL of PMSF per well of a six-well plate. The protein concentration of cell lysates was measured with a BCA protein assay kit (Applygen, Beijing, China). Proteins were separated by sodium dodecyl sulfate-polyacrylamide gel electrophoresis (SDS-PAGE) and electro-transferred to polyvinylidene fluoride (Millipore, Boston, MA, USA) membranes for western blotting. The membrane was blocked with 5% nonfat dry milk (BD, Franklin, LA, USA) for 2 h, and then diluted with primary antibody YTHDF1 (Abcam, Cambridge, UK, 1:1000), E-cadherin (Affinity, Shanghai, China, 1:1000), N-cadherin(Affinity, Shanghai, China, 1:1000), Vimentin(Affinity, Shanghai, China, 1:1000). β-actin (ZSGB-Bio, Beijing, China, 1:1000) was used as the internal control. After overnight incubation, the membranes were washed three times with TBST (supplemented with 0.1% Tween 20) and incubated with HPR-labeled goat anti-rabbit IgG (ZSGB-Bio, Beijing, China, 1:1000) dilution for 2 h at room temperature. After washing with TBST, it was reacted with a luminescent solution (TransGen Biotech, Beijing, China) and developed under a gel imaging system.

### 4.17. Statistical Analysis

Data analyses were obtained with GraphPad Prism 9.0 software (GraphPad, Inc., La Jolla, CA, USA) All experiments were repeated at least three times independently. T-test was used for the difference between the two groups. Statistical analysis was performed in R, and expression differences were visualized with R package ggplot2. The t-test and Mann–Whitney U test were conducted to determine differences between breast cancer tissues and normal paracancerous tissues. ROC curves were performed to examine the area of YTHDF1 expression with the PROC package (* *p* < 0.05; ** *p* < 0.01; *** *p* < 0.001).

## 5. Conclusions

In the current study, m6A regulators are found to be key participants in the malignant evolution of breast cancer. We validated that YTHDF1 expression was elevated in breast cancer tissues. By constructing ceRNA networks, we verified that miR-378g inhibited YTHDF1 expression in breast cancer cells, and miR-378g/YTHDF1 affected the proliferation rate of MDA-MB-231 cells. We propose that YTHDF1 could be used as a prospective biological marker indicating poor prognosis and diagnostic testing in breast cancer. Furthermore, YTHDF1 is predicted to be a promising target in breast cancer immunotherapy since it regulates the ceRNA network and has a specialized function in tumor immunology and breast cancer prognosis.

## Data Availability

Publicly available databases were analyzed in this study. These data can be found here: TCGA website (http://portal.gdc.cancer.gov/, accessed on 23 April 2022), CPTAC (https://proteomics.cancer.gov/programs/cptac, accessed on 4 June 2022), UALCAN (https://ualcan.path.uab.edu/, accessed on 6 June 2022), STRING (http://string-db.org, accessed on 29 May 2022), TIMER (http://cistrome.shinyapps.io/timer/, accessed on 10 June 2022), TISIDB (http://cis.hk/TISIDB/, accessed on 10 June 2022), RMVar (https://rmvar.renlab.org/, accessed on 20 August 2022), SangerBox website (http://sangerbox.com/Tool, accessed on 30 August 2022), RNA22 (https://cm.jefferson.edu/rna22/, accessed on 12 October 2022), TargetScan7 (https://www.targetscan.org/vert_72/, accessed on 17 June 2022), miRDB (https://mirdb.org/, accessed on 26 October 2022), miRNet2.0 (https://www.mirnet.ca/miRNet/home.xhtml, accessed on 27 October 2022), and ENCORI (https://starbase.sysu.edu.cn/index.php, accessed on 27 October 2022).

## Supplementary Materials

The following supporting information can be downloaded at: https://www.mdpi.com/article/10.3390/ijms25031879/s1.
