# Supplementary material for: YTHDF1’s Regulatory Involvement in Breast Cancer Prognosis, Immunity, and the ceRNA Network
Source: Int J Mol Sci. 2024 Feb 4;25(3):1879. doi: 10.3390/ijms25031879 (PMC10856278; doi:10.3390/ijms25031879)
Supplement: Supplementary file 1 [file ijms-25-01879-s001.zip › Table S1.pdf]

Table S1. The nucleotide sequence of Negative control, siNC, siRNA of YTHDF1, and miR378g mimics/inhibitor.

**Name**	**Sequence (5'-3')**
Negative control	UUCUCCGAACGUGUCACGUTT
	ACGUGACACGUUCGGAGAATT
siNC	UUCUCCGAACGUGUCACGUTT
	ACGUGACACGUUCGGAGAATT
siYTHDF1	GCUCAACCGCAGUAUCAGATT
	UCUGAUACUGCGGUUGAGCTT
miR378g mimics	ACUGGGCUUGGAGUCAGAAG
	UCUGACUCCAAGCCCAGUUU
miR378g inhibitor	CUUCUGACUCCAAGCCCAGU
